# Eye gaze is not coded by cardinal mechanisms alone

**DOI:** 10.1098/rspb.2013.1049

**Published:** 2013-08-07

**Authors:** Dominic J. Cheleski, Isabelle Mareschal, Andrew J. Calder, Colin W. G. Clifford

**Affiliations:** 1 School of Psychology, The University of Sydney, Griffith Taylor Building, New South Wales 2006, Australia; 2 Australian Centre of Excellence in Vision Science, The University of Sydney, New South Wales 2006, Australia; 3 School of Psychology, University of New South Wales, Sydney, New South Wales 2052, Australia; 4 Biological and Experimental Psychology, School of Biological and Chemical Sciences, Queen Mary University of London, London, UK; 5 MRC Cognition and Brain Science Unit, Cambridge, UK

**Keywords:** gaze, adaptation, cardinal, non-cardinal

## Abstract

Gaze is an important social cue in regulating human and non-human interactions. In this study, we employed an adaptation paradigm to examine the mechanisms underlying the perception of another's gaze. Previous research has shown that the interleaved presentation of leftwards and rightwards gazing adaptor stimuli results in observers judging a wider range of gaze deviations as being direct. We applied a similar paradigm to examine how human observers encode oblique (e.g. upwards and to the left) directions of gaze. We presented observers with interleaved gaze adaptors and examined whether adaptation differed between congruent (adaptor and test along same axis) and incongruent conditions. We find greater adaptation in congruent conditions along cardinal (horizontal and vertical) and non-cardinal (oblique) directions suggesting gaze is not coded alone by cardinal mechanisms. Our results suggest that the functional aspects of gaze processing might parallel that of basic visual features such as orientation.

## 1. Introduction

Accurately perceiving the direction of another person's eye gaze plays an important social function [1], with evidence linking abnormal gaze behaviour to certain clinical populations (e.g. autism and schizophrenia). Gaze direction communicates information about the mental state, emotion and interest of another individual which can be used to understand the environment and the likely thoughts and future behaviour of that individual (for a review, see [2]). For example, direct gaze can communicate friendliness [3] or threat [2] and averted gaze can communicate avoidance [4] or interest in a particular location in the environment [5].

Most studies, however, have investigated the perception of gaze along a single cardinal axis, the horizontal [6–9]. Much less attention has been devoted in understanding coding mechanisms for vertical and non-cardinal (diagonal) gaze directions. Seminal single cell experiments by Perrett *et al.* [10] revealed that different cells respond to direct gaze and averted gaze in vertical and horizontal directions, respectively, suggesting the existance of channels coding gaze directed towards the left, right, up, down and straight-ahead. Hypothetically, these cardinal dimensions (horizontal and vertical) would be sufficient to encode all eye directions. The existence of more than two (cardinal) channels could provide us with greater sensitivity to detect small deviations in another's gaze, however, it remains an open question as to whether these non-cardinal channels exist.

As far as we are aware, only one study has explored gaze processing where eyes deviate in diagonal directions [11]. Gaze judgements were up to 48 per cent less accurate for gaze in diagonal directions relative to cardinal directions, and there was a tendency to bias diagonal gaze directions towards the cardinal axes [11]. However, it must be noted that these authors used a triadic paradigm involving three entities; a ‘sender’(1) who looked at an object of interest (2) and a ‘receiver’(3) who had to judge which object the sender was looking at. This is contrasted to much of the research on gaze that uses a dyadic paradigm where there are two entities; a ‘sender’ (1), and a receiver (2) who judges the direction of gaze of the ‘sender’ [6,7,10,12,13].

Preferential processing of cardinal directions has also been observed in studies of basic visual attributes, including motion [14,15] and orientation. For example, judgements of the orientation of lines is more accurate near the cardinal axes when compared with the diagonal axes [16]; and when oriented lines are presented under uncertainty, there is a tendency to judge them as tilted towards cardinal directions [17,18]. Thus, it appears there may be something unique about cardinal dimensions in both basic vision and gaze processing (gaze sensitive channels which are tuned along cardinal directions; [11]). Whether in the case of gaze, this reflects an absence of channels coding non-cardinal dimensions remains to be established, however.

Adaptation is a tool frequently used in vision research to examine underlying neural mechanisms (e.g. in colour processing; [19]). Adaptation causes a loss in responsiveness of the mechanisms which code the adapting stimulus [7,20]. A number of studies have examined perceptual changes after gaze adaptation. For example, adapting to interleaved leftwards and rightwards stimuli led to an increase in the range of gaze directions either side of direct gaze categorized as direct (the cone of direct gaze; [9]): i.e. small leftwards and rightwards deviations were more likely judged as being direct following adaptation [6].These effects persisted despite changes in the size of the test face, suggesting that adaptation is not occurring within low-level visual mechanisms that respond to contrast, but rather reflects the adaptation of high-level gaze-processing mechanisms [6]. However, high-level adaptation of face-processing mechanisms has been found to follow a similar time course of build-up and decay as low-level adaptation, suggesting that analogous neural mechanisms might be involved in high- and low-level adaptation [21,22].

In this paper, we used a similar adaptation paradigm to investigate whether only two cardinal mechanisms (horizontal and vertical) exist to encode all gaze deviations. Stimuli were faces with eyes deviated along the horizontal, vertical and two non-cardinal (diagonal) axes: RU/LD (right-up/left-down) and LU/RD (left-up/right-down). Participants adapted to eyes deviated 15° in one direction interleaved with eyes deviated 15° in the opposite direction along the same axis. After adaptation, participants were tested with stimuli along the same axis (congruent trials) or the perpendicular axis (incongruent trials). We investigated the presence of a non-cardinal gaze mechanism by comparing the cone of direct gaze on congruent and incongruent conditions. The reasoning is as follows; after adaptation to gaze on one non-cardinal axis, both cardinal axis mechanisms should be less responsive since adapting along the RU/LD axis adapts left, right, up and down directions. Similarly, adaptation along the LU/RD axis also results in (equal) adaptation of the two cardinal mechanisms. Therefore, if mechanisms exist along cardinal axes only, there should be no difference in the width of the cone of direct gaze between congruent and incongruent conditions along the non-cardinal axes.

## 2. Material and methods

### (a) Apparatus and stimuli

A Dell XPS computer running matlab (MathWorks Ltd) was used for stimulus generation, experiment control and recording subjects' responses. The programs controlling the experiment incorporated elements of the PsychToolbox [23]. Stimuli were displayed on a Sony Trinitron 20SE monitor (1024 × 768 pixels, refresh rate: 75 Hz) driven by the computer's built-in NVIDIA GeForce GTS 240 graphics card. The display was calibrated using a photometer and linearized using look-up tables in software. At the viewing distance of 57 cm, one pixel subtended 2.2 arcmin. Two authors and six naive volunteer subjects (four females in total) aged between 23 and 57 years participated in the study. All participants had normal or corrected to normal vision. All experiments adhered to the Declaration of Helsinki guidelines.

**Figure 1.**
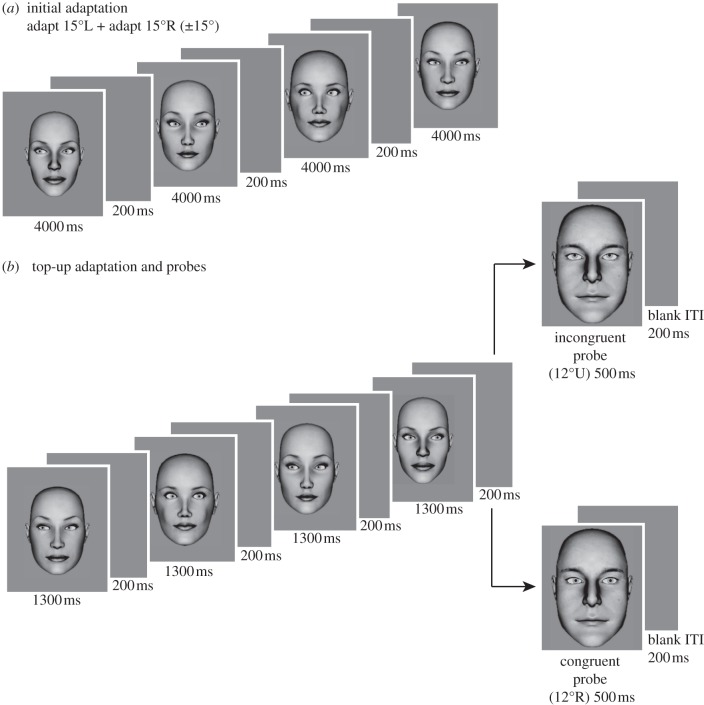
Adaptation and probe stimuli shown along a cardinal (horizontal) axis. (*a*) consists of a series of faces alternating between +15° and −15° gaze direction presented for a total of 1 min. This was immediately followed by (*b*) (the top-up phase) that lasted 6000 ms. At the end of (*b*) a probe face of a different sex and larger size to the adaptation faces was shown and the observer responded using the number keys. Only for illustrative purposes, the figure shows two probes for the two conditions: congruent condition in the bottom (probe is along horizontal axis) and incongruent condition in the top (probe is along vertical axis). L, R, U and D correspond to left, right, up and down, respectively.

Stimuli were grey scale synthetic faces (four males and four females) created with Daz software (http://www.daz3d.com/). The hair was cropped and the face was presented within a circular aperture in the middle of the monitor (see figure 1 for sample face). The stimuli subtended 15.1° × 11.2° and were viewed in a dimly lit room. The original eyes in the faces were replaced using Gimp software by greyscale eye stimuli created using Matlab. The deviation of each eye was then independently controlled using Matlab procedures that gave us precision down to the nearest pixel for eye rotation along any axis.

### (b) Procedure: adaptation

As discussed, adaptation to interleaved stimuli of opposite sign causes a widening in the cone of direct gaze [6]. We sought to determine (i) whether adaptation effects were measurable along the different axes of gaze and (ii) whether adaptation resulted in a wider cone of direct gaze following congruent adaptation compared with incongruent adaptation. Cones of direct gaze were measured in the following order:

— *Pre-adaptation baseline*. The baseline cone of direct gaze along each axis was measured in two separate runs, once using female probe faces and once using male probe faces. Stimuli were presented using a method of constant stimuli with nine different directions of gaze selected from the set: (−12°, −6°, −3°, −1°, 0°, 1°, 3°, 6°, 12°). Each stimulus was presented for 400 ms followed by a grey screen that lasted 600 ms during which no response was recorded. The next trial was only initiated after a response was made following the 600 ms wait period. Timings for the measurement of the pre-adaptation baselines were reduced compared with the study of Calder *et al.* [6] in order to minimize the overall duration of the full experiment. However, we ensured that the inter-stimulus interval (minimum of 600 ms) was long enough to avoid any potential motion or after-image cues. Each direction of gaze was sampled 12 times per run.
— *Adaptation*. This stage involved adapting and testing to congruent and incongruent stimuli for each of the four axes. Between different adaptation conditions, observers took a break of at least 2 h to prevent any carryover adaptation effects. The adaptation was in figure 1. In part figure 1*a*, the subjects adapted to eyes deviated at +15° for 4000 ms (in one direction along the axis tested) followed by a grey screen for 200 ms, then eyes at −15° (the opposite direction along the axis tested) for 4000 ms. This was repeated for each of the four faces (presented at random) for a period of 1 min.
  This was followed immediately by a top-up adaptation and probe (figure 1*b*). In figure 1*b*, four faces (two at +15° and two at −5°) were presented for 1300 ms each, followed by a blank screen for 200 ms and then the probe stimulus presented for 500 ms. The probe stimuli were 33 per cent larger than, and opposite gender to the adapting stimuli to increase their saliency and to minimize the adaptation of low-level mechanisms [6]. There was a 200 ms grey screen wait period following the observer's response. Each probe deviation angle was sampled 12 times in figure 1*b* (four faces × three presentations each). Congruent and incongruent adaptation conditions were run twice separately (once adapting with female faces and adapting with male faces) in a random counterbalanced order across observers.
— *Post-adaptation baseline*. The baseline cones of direct gaze were measured at least 2 h after adaptation, and gender tested was presented in a counterbalanced order to the pre-adaptation sequence.

### (c) Procedure: measuring the cone of direct gaze

The observers' task in each stage was always the same; to indicate whether the direction of gaze in the probe face was direct or averted using number keys. Stimuli were presented along four axes and different number keys to record the observer's response were used accordingly: for the horizontal axis response keys were ‘4’ (left), ‘5’ (direct) and ‘6’ (right), for the vertical axis response keys were ‘2’ (up), ‘5’ and ‘8’ (down), for the LU/RD axis response keys were ‘1’ (left-up), ‘5’ and ‘9’ (right-down) and for the RU/LD axis response keys were ‘3’ (right-up), ‘5’ and ‘7’ (left-down). These different axes correspond to stimuli whose deviations were along the horizontal, vertical, oblique at +45° (RU/LD) or oblique at −45° (LU/RD).

Each observer's data within the same condition and axis were compiled across runs and logistic functions were fitted to the proportion of ‘left’ and ‘right’ responses (for the horizontal axis). A function for ‘direct’ responses was calculated by subtracting the sum of the ‘left’ and ‘right’ responses from one. These three functions were fitted as an ensemble using the Nelder–Mead simplex method [24] implemented via Matlab's fminsearch function to minimize residual variance. The separation between the cross-over points of the ‘direct’ and the ‘left’, and ‘direct’ and ‘right’ responses, respectively, is taken as the cone of direct gaze. A similar procedure was employed along the three other axes, plotting the proportion of RU/LD axis; LU/RD axis or ‘down’ and ‘up’ (vertical axis) responses.

### (d) Statistical analysis

Effect sizes are reported using Cohen's *d* for 1 d.f. tests [25] and partial eta-squared 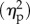 for all other tests.

Data are available from the University of Sydney, Clifford Lab.

## 3. Results

### (a) Cardinal axes: left/right and up/down

**Figure 2.**
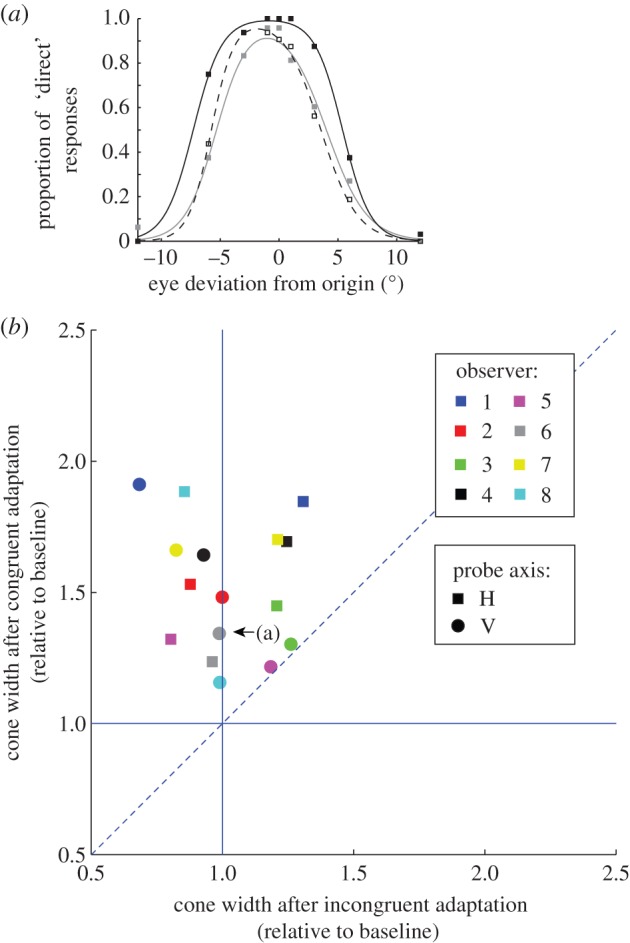
Adaptation effects on cone of direct gaze for congruent and incongruent conditions with adaptation and testing on cardinal axes. (*a*) Cones of direct gaze measured in observer 6 along the vertical axis under three different adaptation conditions (congruent, solid lines; incongruent, dashed lines; baseline, grey lines). (*b*) Adaptation data for all observers. Congruent and incongruent cones of direct gaze were divided by average baseline (average of pre- and post-adaptation baselines). The dotted line represents a line of equality where congruent and incongruent adaptations have the same effect on cone of direct gaze. The vertical and horizontal solid lines represent lines of equality where incongruent and congruent adaptation, respectively, do not vary from baseline cone of direct gaze. Point labelled (a) represents the data in the top panel.

The cone of direct gaze was measured along cardinal axes under four conditions and widths are reported in table 1. Figure 2*a* plots the cones of direct gaze for one naive observer (O6) along the vertical axis after congruent adaptation (solid curve), incongruent adaptation (dashed curve) and in the average baseline (pre- and post-adaptation baselines: grey curve). This observer shows selective effects of adaptation: the cone of direct gaze in the congruent condition is wider than baseline, whereas incongruent adaptation only moderately increased the cone of direct gaze.

**Table 1.** Mean cone of direct gaze when adapting and testing along cardinal axes. Note. H and V stand for the horizontal and vertical axes, respectively. Values are the mean width in degrees of visual angle, standard deviations of the mean in brackets.

	adapt H	adapt V
probe H	9.56° (2.17°)	6.41° (1.72°)
probe V	7.33° (2.75°)	10.48° (1.99°)

Figure 2*b* plots the cone of direct gaze after congruent adaptation (divided by baseline) against the cone of direct gaze after incongruent adaptation (divided by baseline) for all observers. Data points above the line of equality (dotted line) represent conditions where congruent adaptation resulted in a larger cone of direct gaze than incongruent adaptation. All the data for the cardinal axes fall above this line. Using a 2 × 2 repeated measures ANOVA, we find that the cone of direct gaze did not vary according to adapting axis (averaged over probe axis conditions), *F*_1,7_ < 0.01, *p* > 0.9, *d* < 0.01, or according to probe axis (averaged over adapting axis conditions) *F*_1,7_ = 2.17, *p* > 0.15, *d* = 0.52. Congruent adaptation (adapt/probe horizontal or adapt/probe vertical) resulted in a significantly wider cone of direct gaze than incongruent adaptation (adapt horizontal/probe vertical or adapt vertical/probe horizontal), on average, *F*_1,7_ = 42.26, *p* < 0.01, *d* = 2.30. Tests of simple effects revealed that the cone of direct gaze was significantly wider after congruent adaptation relative to incongruent adaptation when the probe was horizontal, *F*_1,7_ = 27.75, *p* < 0.01, *d* = 1.86, and when the probe was vertical, *F*_1,7_ = 13.38, *p* < 0.01, *d* = 1.29**.**

### (b) Non-cardinal axes: left-up/right-down and right-up/left-down

**Figure 3.**
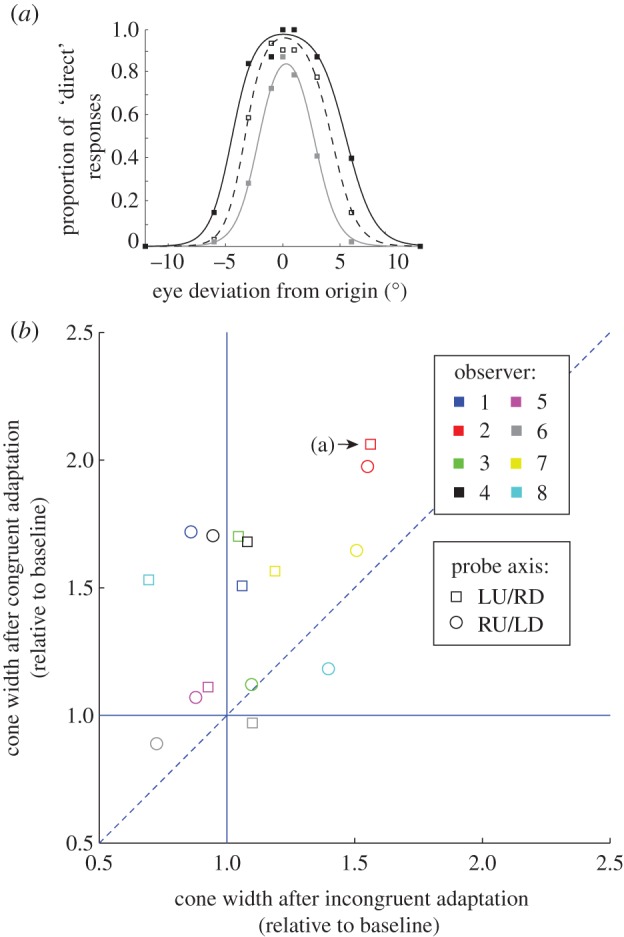
Adaptation effects on the cone of direct gaze for congruent and incongruent conditions with adaptation and testing on non-cardinal axes. (*a*) Cones of direct gaze measured in observer 2 along the LU/RD axis under three different adaptation conditions (congruent, solid lines; incongruent, dashed lines; baseline, grey lines). (*b*) Adaptation data for all observers. Congruent and incongruent cones of direct gaze were divided by average baseline (average of pre- and post-adaptation baselines). The dotted line represents a line of equality where congruent and incongruent adaptations have the same effect on the cone of direct gaze. The vertical and horizontal solid lines represent lines of equality where incongruent and congruent adaptation, respectively, do not vary from baseline cone of direct gaze. Point labelled (a) represents the data in the top panel.

Table 2 reports the cone of direct gaze along non-cardinal axes measured in four conditions. Figure 3*a* plots data for one of the authors (O2) on the LU/RD probe axis, who showed a larger cone of direct gaze for congruent adaptation relative to incongruent adaptation.

**Table 2.** Mean cone of direct gaze when adapting and testing along oblique axes. Note, LU/RD stands for the left and up to right and down axis. RU/LD stands for the right and up to left and down axis. Values are the mean width in degrees of visual angle, standard deviations of the mean in brackets.

	adapt LU/RD	adapt RU/LD
probe LU/RD	10.16° (2.57°)	7.25° (1.84°)
probe RU/LD	7.61° (2.64°)	9.29° (1.62°)

Using a 2 × 2 repeated measures ANOVA, we find that the cone of direct gaze did not significantly differ according to adapting axis (LU/RD; RU/LD, averaged over probe axis conditions), *F*_1,7_ = 0.67 *p* > 0.4, *d* = 0.29, or according to probe axis (LU/RD; RU/LD, averaged over adapting axis conditions) *F*_1,7_ = 0.66, *p* > 0.4, *d* = 0.29. Congruent adaptation (adapt/probe (LU/RD) or adapt/probe (RU/LD)) resulted in a significantly larger cone of direct gaze than incongruent adaptation (adapt (LU/RD)/probe (RU/LD) or adapt (RU/LD)/probe (LU/RD)), on average, *F*_1,7_ = 24.11, *p* < 0.01, *d* = 1.73. This can be visualized in figure 3*b*, where all but two data points for the non-cardinal axes fall above the line of equality (dotted line). Tests of simple effects revealed that the cone of direct gaze was significantly wider after congruent adaptation relative to incongruent adaptation when the probe was LU/RD, *F*_1,7_ = 9.14, *p* < 0.02, *d* = 1.07, but narrowly missed significance when the probe stimulus was RU/LD, *F*_1,7_ = 4.32, *p* = 0.076, *d* = 0.73.

### (c) Adaptation relative to baseline

To determine whether adaptation changed the size of the cone of direct gaze, we compared average baselines with adaptation data in a 4 × 3 repeated measures ANOVA examining the effects of axis of the probe (horizontal, vertical, LU/RD and RU/LD) and adaptation condition (congruent adaptation, incongruent adaptation and no adaptation); the no adaptation condition was the average of pre- and post-baselines (see last column in table 3). There was no significant difference in the cone of direct gaze according to the axis tested, averaged across adaptation conditions, *F*_3,21_ = 1.68, *p* > 0.2, 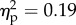.

**Table 3.** Mean cone of direct gaze for baselines measured before (pre) and after (post) adaptation. Note, H, V, LU/RD, RU/LD stands for the horizontal, vertical, left and up to right and down, and right and up to left and down axes, respectively. Means are in degrees of visual angle, standard deviation of the mean shown in brackets.

axis	pre	post	average
H	6.86° (2.82°)	5.28° (0.88°)	6.15° (1.76°)
V	8.31° (3.45°)	6.51° (1.11°)	7.33° (1.77°)
LU/RD	7.15° (2.91°)	6.71° (1.92°)	6.91° (2.01°)
RU/LD	7.49° (3.11°)	6.55° (1.74°)	7.00° (2.20°)

There was a significant effect of adaptation condition on the cone of direct gaze, averaged across axes (*F*_2,14_ = 36.31, *p* < 0.001, 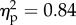), but the interaction was not significant suggesting this effect did not differ according to axis tested, *F*_6,42_ = 0.65, *p* > 0.6, 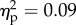. Contrasts revealed the cone of direct gaze was significantly larger after congruent adaptation relative to mean baseline, averaged across the different axes, *F*_1,7_ = 42.23, *p* < 0.001, *d* = 1.07, and when the horizontal (*F*_1,7_ = 48.34, *p* < 0.001, *d* = 2.46), vertical (*F*_1,7_ = 40.98, *p* < 0.001, *d* = 2.26), LU/RD (*F*_1,7_ = 20.11, *p* < 0.001, *d* = 1.58) and RU/LD (*F*_1,7_ = 9.23, *p* = 0.019, *d* = 1.07) axes were analysed separately. Data points in figures 2*b* and 3*b* above horizontal solid line represent occurrences where congruent adaptation resulted in a larger cone of direct gaze relative to baseline. Note that all but two data points (shown in figure 3*b*) are above the horizontal line.

Furthermore, there was no significant difference in the cone of direct gaze when incongruent adaptation and baseline conditions were compared, averaged over all axes (*F*_1,7_ = 1.35, *p* > 0.2, *d* = 0.41), and when the horizontal (*F*_1,7_ = 0.43, *p* > 0.5, *d* = 0.23), vertical (*F*_1,7_ < 0.01, *p* > 0.9, *d* < 0.01), LU/RD (*F*_1,7_ = 0.34, *p* > 0.5, *d* = 0.21) and RU/LD (*F*_1,7_ = 0.058, *p* > 0.4, *d* = 0.27) axes were analysed separately. In figures 2*b* and 3*b*, the vertical (solid) line represents conditions where incongruent adaptation is not different to baseline. The data are clustered around the vertical solid line in both figures indicating incongruent adaptation did not result in a different cone of direct gaze relative to baseline. Overall, the cone of direct gaze was increased for the congruent but not incongruent conditions relative to baseline for both the horizontal and vertical axes and for both the non-cardinal axes.

### (d) Pre- and post-adaptation baselines

**Figure 4.**
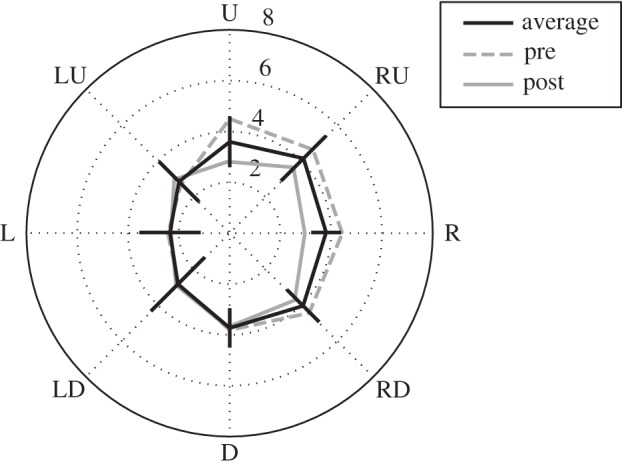
Baselines measured before (pre) and after (post) adaptation (with an average of pre and post). R, L, U, D, RU, LU, RD, LD correspond to right, left, up, down, right and up, left and up, right and down, left and down data points, respectively. The cone of direct gaze is represented by the distance between two points on an axis: L and R (horizontal) or U and D (vertical) or LU and RD or RU and LD. Numbers correspond to the deviation of the stimuli eyes from the origin in degrees of visual angle. Error bars represent the 95% confidence interval of the mean for the average of pre- and post-baselines.

Figure 4 plots the pre- and post-adaptation baselines, averaged across all observers (values in table 3). Pre- and post-adaptation baselines (averaged over axis) were not significantly different when compared in a (4 × 2) repeated measures ANOVA, *F*_1,7_ = 1.91, *p* > 0.05, *d* = 0.49. The interaction was not significant, *F*_3,21_ = 0.9, *p* > 0.4, 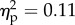. However, there were significant differences in the cone of direct gaze between different axes, averaged over time of testing, *F*_3,21_ = 3.50, *p* = 0.034, 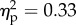. The cone of direct gaze on the horizontal was narrower than the vertical, *F*_1,7_ = 6.51, *p* = 0.038, *d* = 0.90, and the non-cardinal axes (on average), *F*_1,7_ = 6.14 *p* = 0.042, *d* = 0.88 which did not significantly differ in size, *F*_1,7_ = 0.09, *p* > 0.7, *d* = 0.10, averaged over time of testing. The cone of direct gaze was not significantly different when the vertical axis was compared with the non-cardinal axes (on average), *F*_1,7_ = 1.44, *p* > 0.2, *d* =0.35, averaged over time of testing.

## 4. Discussion

We find that adaptation is greater in congruent conditions compared with incongruent conditions for stimuli presented on cardinal and non-cardinal axes. We also report that congruent, but not incongruent, adaptation results in a significantly wider cone of direct gaze relative to baseline. Finally, in the absence of adaptation (baseline) the cones of direct gaze on the two non-cardinal axes and the vertical axes were similar in size and all significantly wider than the cone measured on the horizontal axis.

Our finding that adaptation along non-cardinal axes depends on adaptor-test congruence supports the existence of at least one non-cardinal mechanism that codes gaze. This is because adapting along either non-cardinal axis should have the same effect on cardinal mechanisms, such that if gaze is coded in terms of cardinal mechanisms alone, congruent and incongruent adaptation along non-cardinal axes would result in cones of direct gaze of a similar width. Instead our data indicate the existence of at least one channel tuned to gaze along an oblique axis. It is important to note that we do not refute that cardinal axes may play a unique role in gaze (as suggested by [11]), simply that gaze is not coded exclusively by these cardinal mechanisms.

As mentioned previously, all gaze directions could in principle be sufficiently be coded by two cardinal mechanisms. However, the results here indicate there must be at least one non-cardinal mechanism coding for gaze. There are many possible configurations for processing gaze directions with the presence of a gaze mechanism operating along a non-cardinal axis. For example, there may be one cardinal and one non-cardinal mechanism, or a configuration involving three or more mechanisms. Presumably, a greater number of mechanisms would lead to an enhanced ability to resolve gaze directions accurately.

FMRI adaptation in humans [26] and previous electrophysiological recordings in monkeys [10] have revealed specific neural mechanisms that respond to different directions of gaze along the vertical and horizontal axes (in heads that were either forward facing or rotated). Yet, these experiments have not explored the specific angular tuning of such neurons. For example, a neuron responding to a ‘left averted’ gaze might actually have a preferred direction of ‘left and slightly up’. More detailed physiological experimentation is needed to determine the relative prevalence and properties of cardinal and non-cardinal gaze sensitive cells. It is possible that the distribution of gaze neurons may mirror that seen in the coding of orientation, where there is a continuum of mechanisms tuned to different orientations, with a greater prevalence of mechanisms that code in cardinal directions [27–29]. Indeed, there is already evidence that cells tuned to head orientation are more likely to exhibit a preference for cardinal head orientations (full face, back of head and left and right profiles) than intermediate orientations [30]. As far as we are aware, however, this has not been addressed for gaze direction.

An analysis of the baseline data reveals that a similar range of eye gaze deviations are categorized as direct along the vertical and two non-cardinal axes (oriented at +45° and −45° to the horizontal), while a more narrow range of eye gaze deviations are categorized as direct along the horizontal (figure 4). This is consistent with Vida & Maurer [13], who report narrower cone of direct gaze on the horizontal axis relative to the vertical axis. Additionally, our estimates of the cone of direct gaze along the horizontal (and vertical) axis, 6.15° (and 7.33°), are similar to earlier estimates, 5.49° (and 6.96°, [13]) and 5.6° [31].

Specific gaze directions can communicate valuable social information. For example, direct gaze can indicate approach emotions (in the subject of interest) such as anger and joy, whereas averted gaze can indicate avoidance emotions such as sadness and fear [32]. Looking up and slightly averted to one side can indicate the subject is thinking [33], and averted gaze can also elicit mistrust in the observer [34]. Our results may bear on certain clinical populations who display an abnormal ability to represent and/or interpret the social meaning of specific gaze directions, such as people with autism [35–37], schizophrenia [38], social phobia [39], Turner's syndrome [40] and William's syndrome [41]. For example, school-aged children with autism are reported to perform more poorly than typical children when asked to make fine-grained judgements on gaze direction [35] or to identify the target of another's gaze [42]. Since additional gaze mechanisms could increase the ability to make accurate judgements of gaze direction, the reduced ability to make accurate judgements of gaze direction in school-aged children with autism might be accounted for by the absence of one or more non-cardinal mechanisms for gaze.

People with autism have also been shown to lack normal adaptive mechanisms coding for social stimuli such as facial identity [43] and gaze [37]. It has been suggested that these results are consistent with a deficit in incorporating prior knowledge into the processing of such social stimuli [44]. It has recently been shown that normal adult observers have a prior expectation that another's gaze is directed towards them [45]. However, the findings of Pellicano *et al.* [37] and Mareschal *et al.* [45] are all based on stimuli in which gaze varied along the horizontal. Future work using non-cardinal stimuli such as those employed here would allow a fuller characterization of the normally functioning gaze-processing system and the deficits associated with autism spectrum disorder.

## References

[1] Baron-Cohen S Campbell R Karmiloff-Smith A Grant J Walker J 1995 Are children with autism blind to the mentalistic significance of the eyes? Br. J. Dev. Psychol. 13, 379–398 10.1111/j.2044-835X.1995.tb00687.x (doi:10.1111/j.2044-835X.1995.tb00687.x)

[2] Emery N 2000 The eyes have it: the neuroethology, function and evolution of social gaze. Neurosci. Biobehav. Rev. 24, 581–604 10.1016/S0149-7634(00)00025-7 (doi:10.1016/S0149-7634(00)00025-7) 10940436

[3] Brothers L Ring B Kling A 1990 Response of neurons in the macaque amygdala to complex social stimuli. Behav. Brain Res. 41, 199–213 10.1016/0166-4328(90)90108-Q (doi:10.1016/0166-4328(90)90108-Q) 2288672

[4] Hietanen JK Peltola MJ Linna-aho K Ruuhiala HJ 2008 Seeing direct and averted gaze activates the approach-avoidance motivational brain systems. Neuropsychologia 46, 2423–2430 10.1016/j.neuropsychologia.2008.02.029 (doi:10.1016/j.neuropsychologia.2008.02.029) 18402988

[5] Itier RJ Batty M 2009 Neural bases of eye and gaze processing: the core of social cognition. Neurosci. Biobehav. Rev. 33, 843–863 10.1016/j.neubiorev.2009.02.004 (doi:10.1016/j.neubiorev.2009.02.004) 19428496 PMC3925117

[6] Calder AJ Jenkins R Cassel A Clifford CWG 2008 Visual representation of eye gaze is coded by a nonopponent multichannel system. J. Exp. Psychol. Gen. 137, 244–261 10.1037/0096-3445.137.2.244 (doi:10.1037/0096-3445.137.2.244) 18473657

[7] Jenkins R Beaver JD Calder AJ 2006 I thought you were looking at me direction-specific aftereffects in gaze perception. Psychol. Sci. 17, 506–513 10.1111/j.1467-9280.2006.01736.x (doi:10.1111/j.1467-9280.2006.01736.x) 16771801

[8] Seyama J Nagayama RS 2006 Eye direction aftereffect. Psychol. Res. 70, 59–67 10.1007/s00426-004-0188-3 (doi:10.1007/s00426-004-0188-3) 15378364

[9] Gamer M Hecht H 2007 Are you looking at me? Measuring the cone of gaze. J. Exp. Psychol. Hum. Percep. Perform. 33, 705–715 10.1037/0096-1523.33.3.705 (doi:10.1037/0096-1523.33.3.705) 17563231

[10] Perrett D Smith P Potter D Mistlin A Head A Milner A Jeeves M 1985 Visual cells in the temporal cortex sensitive to face view and gaze direction. Proc. R. Soc. Lond. B 223, 293–317 10.1098/rspb.1985.0003 (doi:10.1098/rspb.1985.0003) 2858100

[11] Bock SW Dicke P Thier P 2008 How precise is gaze following in humans? Vis. Res. 48, 946–957 10.1016/j.visres.2008.01.011 (doi:10.1016/j.visres.2008.01.011) 18294671

[12] Cline MG 1967 The perception of where a person is looking. Am. J. Psychol. 80, 41–50 10.2307/1420539 (doi:10.2307/1420539) 6036357

[13] Vida MD Maurer D 2012 The development of fine-grained sensitivity to eye contact after 6 years of age. J. Exp. Child Psychol. 112, 243–256 10.1016/j.jecp.2012.02.002 (doi:10.1016/j.jecp.2012.02.002) 22417921

[14] Ball K Sekuler R 1987 Direction-specific improvement in motion discrimination. Vis. Res. 27, 953–965 10.1016/0042-6989(87)90011-3 (doi:10.1016/0042-6989(87)90011-3) 3660656

[15] Gros BL Blake R Hiris E 1998 Anisotropies in visual motion perception: a fresh look. JOSA A 15, 2003–2011 10.1364/JOSAA.15.002003 (doi:10.1364/JOSAA.15.002003) 9691484

[16] Appelle S 1972 Perception and discrimination as a function of stimulus orientation: the ‘oblique effect’ in man and animals. Psychol. Bull. 78, 266–278 10.1037/h0033117 (doi:10.1037/h0033117) 4562947

[17] Tomassini A Morgan MJ Solomon JA 2010 Orientation uncertainty reduces perceived obliquity. Vision Res. 50, 541–547 10.1016/j.visres.2009.12.005 (doi:10.1016/j.visres.2009.12.005) 20005889

[18] Girshick AR Landy MS Simoncelli EP 2011 Cardinal rules: visual orientation perception reflects knowledge of environmental statistics. Nat. Neurosci. 14, 926–932 10.1038/nn.2831 (doi:10.1038/nn.2831) 21642976 PMC3125404

[19] Webster MA Mollon J 1991 Changes in colour appearance following post-receptoral adaptation. Nature 349, 235–238 10.1038/349235a0 (doi:10.1038/349235a0) 1987475

[20] Webster MA 2011 Adaptation and visual coding. J. Vis. 11, 3 10.1167/11.5.3 (doi:10.1167/11.5.3) 21602298 PMC3245980

[21] Leopold DA Rhodes G Muller KM Jeffrey L 2005 The dynamics of visual adaptation to faces. Proc. R. Soc. B 272, 897–904 10.1098/rspb.2004.3022 (doi:10.1098/rspb.2004.3022) PMC1564098 16024343

[22] Rhodes G Jeffrey L Clifford CW Leopold DA 2007 The timecourse of higher-level face aftereffects. Vis. Res. 17, 2291–2296 10.1016/j.visres.2007.05.012 (doi:10.1016/j.visres.2007.05.012) 17619045

[23] Brainard DH 1997 The psychophysics toolbox. Spatial Vis. 10, 433–436 9176952

[24] Nelder JA Mead R 1965 A simplex method for function minimization. Comp. J. 7, 308–313 10.1016/j.visres.2007.05.012 (doi:10.1016/j.visres.2007.05.012)

[25] Cohen J 1988 Statistical power analysis for the behavioral sciences. Lawrence Erlbaum

[26] Calder AJ Beaver JD Winston JS Dolan RJ Jenkins R Eger E Henson RNA 2007 Separate coding of different gaze directions in the superior temporal sulcus and inferior parietal lobule. Curr. Biol. 17, 20–25 10.1016/j.cub.2006.10.052 (doi:10.1016/j.cub.2006.10.052) 17208181 PMC1885952

[27] Chapman B Bonhoeffer T 1998 Overrepresentation of horizontal and vertical orientation preferences in developing ferret area 17. Proc. Natl Acad. Sci. USA 95, 2609–2614 10.1073/pnas.95.5.2609 (doi:10.1073/pnas.95.5.2609) 9482934 PMC19431

[28] De Valois RL William YE Hepler N 1982 The orientation and direction selectivity of cells in macaque visual cortex. Vis. Res. 22, 531–544 10.1016/0042-6989(82)90112-2 (doi:10.1016/0042-6989(82)90112-2) 7112953

[29] Li B Peterson MR Freeman RD 2003 Oblique effect: a neural basis in the visual cortex. J. Neurophysiol. 90, 204–217 10.1152/jn.00954.2002 (doi:10.1152/jn.00954.2002) 12611956

[30] Perrett DI Oram MW Harries MH Bavan R Hietanen JK Benson PJ Thomas SM 1991 Viewer-centred and object-centred coding of heads in the macaque temporal cortex. Exp. Brain Res. 86, 159–173 10.1007/BF00231050 (doi:10.1007/BF00231050) 1756786

[31] Gibson JJ Pick AD 1963 Perception of another person's looking behavior. Am. J. Psychol. 76, 386–394 10.2307/1419779 (doi:10.2307/1419779) 13947729

[32] Adams RB Kleck RE 2005 Effects of direct and averted gaze on the perception of facially communicated emotion. Emotion 5, 3–11 10.1037/1528-3542.5.1.3 (doi:10.1037/1528-3542.5.1.3) 15755215

[33] Baron-Cohen S Cross P 1992 Reading the eyes: evidence for the role of perception in the development of a theory of mind. Mind Lang. 7, 172–186 10.1111/j.1468-0017.1992.tb00203.x (doi:10.1111/j.1468-0017.1992.tb00203.x)

[34] Einav S Hood BM 2008 Tell-tale eyes: children's attribution of gaze aversion as a lying cue. Dev. Psychol. 44, 1655–1667 10.1037/a0013299 (doi:10.1037/a0013299) 18999328

[35] Campbell R Lawrence K Mandy W Mitra C Jeyakuma L Skuse D 2006 Meanings in motion and faces: developmental associations between the processing of intention from geometrical animations and gaze detection accuracy. Dev. Psychopathol. 18, 99–118 10.1017/S0954579406060068 (doi:10.1017/S0954579406060068) 16478554

[36] Webster S Potter DD 2008 Eye direction detection improves with development in autism. J. Autism Dev. Disord. 38, 1184–1186 10.1007/s10803-008-0539-9 (doi:10.1007/s10803-008-0539-9) 18324465

[37] Pellicano E Rhodes G Calder AJ 2013 Reduced gaze aftereffects are related to difficulties categorizing gaze direction in autism. Neuropsychologia 51, 1504–1509 10.1016/j.neuropsychologia.2013.03.021 (doi:10.1016/j.neuropsychologia.2013.03.021) 23583965 PMC3708125

[38] Tso IF Mui ML Taylor SF Deldin PJ 2012 Eye-contact perception in schizophrenia: relationship with symptoms and socioemotional functioning. J. Abnorm. Psychol. 121, 616–627 10.1037/a0026596 (doi:10.1037/a0026596) 22250658

[39] Gamer M Hecht H Seipp N Hiller W 2011 Who is looking at me? The cone of gaze widens in social phobia. Cogn. Emotion 25, 756–764 10.1080/02699931.2010.503117 (doi:10.1080/02699931.2010.503117) 21547777

[40] Elgar K Campbell R Skuse D 2002 Are you looking at me? Accuracy in processing line–of–sight in Turner syndrome. Proc. R. Soc. Lond. B 269, 2415–2422 10.1098/rspb.2002.2173 (doi:10.1098/rspb.2002.2173) PMC1691184 12495483

[41] Mobbs D Garrett A Menon V Rose F Bellugi U Reiss AL 2004 Anomalous brain activation during face and gaze processing in Williams syndrome. Neurology 62, 2070–2076 10.1212/01.WNL.0000129536.95274.DC (doi:10.1212/01.WNL.0000129536.95274.DC) 15184616

[42] Frischen A Bayliss AP Tipper SP 2007 Gaze cueing of attention: visual attention, social cognition, and individual differences. Psychol. Bull. 133, 694–724 10.1037/0033-2909.133.4.694 (doi:10.1037/0033-2909.133.4.694) 17592962 PMC1950440

[43] Pellicano E Jeffery L Burr D Rhodes G 2007 Abnormal adaptive face-coding mechanisms in children with autism spectrum disorder. Curr. Biol. 17, 1508–1512 10.1016/j.cub.2007.07.065 (doi:10.1016/j.cub.2007.07.065) 17764946

[44] Pellicano E Burr D 2012 When the world becomes ‘too real’: a Bayesian explanation of autistic perception. Trends Cogn. Sci. 16, 504–510 10.1016/j.tics.2012.08.009 (doi:10.1016/j.tics.2012.08.009) 22959875

[45] Mareschal I Calder AJ Clifford CW 2013 Humans have a prior expectation that gaze is directed toward them. Curr. Biol. 23, 717–721 10.1016/j.cub.2013.03.030 (doi:10.1016/j.cub.2013.03.030) 23562265 PMC3918857

